# TLP-CCC: Temporal Link Prediction Based on Collective Community and Centrality Feature Fusion

**DOI:** 10.3390/e24020296

**Published:** 2022-02-20

**Authors:** Yuhang Zhu, Shuxin Liu, Yingle Li, Haitao Li

**Affiliations:** Institute of Information Technology, PLA Strategic Support Force Information Engineering University, Zhengzhou 450002, China; zyh@ndsc.com.cn (Y.Z.); lyl@ndsc.com.cn (Y.L.); lht@ndsc.com.cn (H.L.)

**Keywords:** temporal link prediction, collective influence, community detection, random walk, representation learning, multi feature fusion

## Abstract

In the domain of network science, the future link between nodes is a significant problem in social network analysis. Recently, temporal network link prediction has attracted many researchers due to its valuable real-world applications. However, the methods based on network structure similarity are generally limited to static networks, and the methods based on deep neural networks often have high computational costs. This paper fully mines the network structure information and time-domain attenuation information, and proposes a novel temporal link prediction method. Firstly, the network collective influence (CI) method is used to calculate the weights of nodes and edges. Then, the graph is divided into several community subgraphs by removing the weak link. Moreover, the biased random walk method is proposed, and the embedded representation vector is obtained by the modified Skip-gram model. Finally, this paper proposes a novel temporal link prediction method named TLP-CCC, which integrates collective influence, the community walk features, and the centrality features. Experimental results on nine real dynamic network data sets show that the proposed method performs better for area under curve (AUC) evaluation compared with the classical link prediction methods.

## 1. Introduction

Link prediction method has been applied to community detection, anomaly detection, influence analysis, and recommendation systems for complex networks. With network topology, attributes, and network time series evolution information, link prediction aims to solve one of the most basic scientific problems, reconstructing and predicting missing information [1,2]. Specifically, link prediction research includes edge missing, edge anomaly, and possible future connections in the network [3,4]. In addition to its application value, the related technology of link prediction has important theoretical research value. It can provide a reasonable explanation for the network evolution mechanism, mine the law of network dynamic changes, and provide reliable theoretical support for understanding the mechanism of network internal changes [5]. Complex networks in the real world are often dynamic and temporal networks, nodes and edges in the network change continuously with time. The method of temporal link prediction can better mine the historical information of network changes, and it can be more effective to achieve the prediction performance.

In recent years, link prediction has many research results in large-scale networks, multidimensional heterogeneous networks, and dynamic temporal networks. Generally, the prediction methods can be divided into three categories: network structure-based methods, likelihood analysis-based methods, and machine learning-based methods [6]. Different categories of prediction methods are based on different network scenarios. The network structure-based methods include structure similarity methods such as Common Neighbors [7], Adamic-Adar [8], Local path [9], Katz [10], etc. They only utilize network connection information and have the widest application scenarios. The method based on likelihood analysis uses the known topology and attribute information to calculate the probability of nonexistent edges. For example, Zhao et al. [11] proposed a Bayesian probability model, which combines the node attributes in various directed and undirected relational networks. Liu Shuxin et al. [12] proposed a similarity model based on the matching degree of bidirectional transmission of resources. Javari et al. [13] establishes link label models for local and global attributes of sparse networks to achieve prediction-related functions. Pan et al. [14] combines clustering mechanisms to propose a conditional probability model of the closed paths. Although these methods can make good use of topological structure information, the computational complexity of the algorithms is high and is not applicable for large-scale networks; machine learning methods have been studied in recent years. The idea is to input the structure and attribute information of the network into various neural networks for training, output the embedded vector representation of nodes, and realize the functions of classification and prediction. Li et al. [15] uses the deep learning method optimized by a limited Boltzmann machine to achieve dynamic network link prediction. With the long short-term memory network embedding time information and graph neural network embedding structure information, Chen et al. [16] shows that the combined vectors greatly improve the accuracy of prediction. Machine learning methods usually perform better than similarity-based methods and have lower time complexity than likelihood probability-based models. However, in real applications, the machine learning methods often need more harsh conditions, and the training process of the optimal parameters needs to consume more resources.

Currently, with the popularity of social media, more and more researchers use classical sociological theory to study link prediction problems in social networks [17,18]. Liu Shuxin et al. [19] regards the motif as the smallest community and defines the three-tuple community consistency index to describe the impact of the three-tuple community attributes on link prediction. Valverde-Rebaza et al. [20] proposed a prediction method based on the combination of user interest behavior and community information. Liu et al. [21] proposed a link prediction method based on weak links, degree, and betweenness of common neighbors. Although simple use of social information improves prediction accuracy, these methods do not distinguish the different effects of different community sizes on prediction results, and can further explore the impact of different community structures on similarity.

Existing community theory and structural similarity methods often solve link prediction problems on static networks, but few on dynamic networks. Aiming at the link prediction problem of dynamic networks, this paper proposes a temporal link prediction approach based on community multi-feature fusion and embedded representation, which combines the methods of influence optimization, community detection, and network embedded representation based on the network topology information. The main contributions include:

- Motivated by the concept of collective influence in percolation optimization theory, the collective influence is considered as the effective attribute of nodes, and construct the weight matrix of edges based on node attribute.
- With CI-based weight matrix and weak link optimization, we use the Louvain algorithm to partition the dynamic network into communities, and design the mechanism of node random walk within the community.
- Different from Deepwalk and node2vec, we design a novel strategy of next hop with priority to the existence of connected edges, and the improved Skip-gram model is used to obtain the node representation vector.
- Concatenate the collective influence, network centrality impact and the representation learning vectors of nodes. By using the joint new vectors to calculate the score matrix of the edges, and the temporal link prediction method TLP-CCC is proposed.
- The experimental results on nine real dynamic network data sets show that the proposed method outperforms the traditional classical temporal link prediction methods under AUC evaluation metric.

## 2. Preliminary

### 2.1. Temporal Network

**Figure 1:**
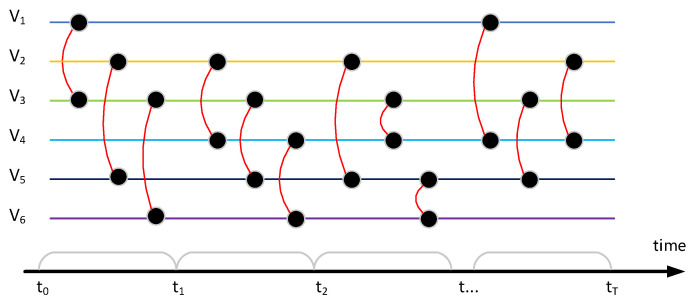
Schematic diagram of temporal link prediction. Edges$\left(e_{13},e_{25},e_{36}\right)$ exist in a range of $\left[t_{0},t_{1}\right]$, edges$\left(e_{24},e_{35},e_{46}\right)$ exist in a range of $\left[t_{1},t_{2}\right]$, edges$\left(e_{25},e_{34},e_{56}\right)$ exist in a range of $\left[t_{2},t_{3}\right]$, edges$\left(e_{14},e_{35},e_{24}\right)$ exist in a range of $\left[t_{T-1},t_{T}\right]$. The edges are discretized into a snapshot list [1,…,T].

The temporal network includes continuous and discrete temporal network models, and most of them are discrete temporal. As shown in Figure 1, due to the temporal nature of network evolution, a fixed discrete-time window is set to discretize the continuous activities in different time windows. For example, user $V_{1}$ interacts with user $V_{3}$ in window $t_{1}$. The discrete-temporal model does not consider the situation that the communication continues into the next window; it is considered that the interaction only exists inside the $t_{1}$ window. In a window unit, repeated multiple contacts are only considered one time, and the node’s own connection is not considered. At the same time, the sampling measurement of the network is set between each time window, so that the network topology in a window period before sampling forms a snapshot diagram.

Given a temporal network *G* that has *N* nodes and *E* edges, each edge in set *E* can be expressed as e(i,j), indicating that node *i* and node *j* have a connection relationship in the time window *t*. If the continuous time is discretized according to the fixed time interval *w*, the window range generated by the edge connection can be expressed as [t−w,t).

### 2.2. Problem Definition

Given a dynamic network *G*, it is divided into *T* network snapshot sequences $G=\{G_{1},G_{2},\cdots ,G_{T})$ by fixed time intervals, where $G_{t}=G\left(V_{t},E_{t}\right)$ represents the network snapshot at time *t*, $V_{t}$ is the node set and $E_{t}$ is the edge set at time *t*. Because this paper focuses on the link prediction problem, we only consider the change of edge connection with time, and fix the node set at different times as *V*. The adjacency matrix of network snapshot at each time *t* can be expressed as $A_{t}={\left[a_{ij}^{t}\right]}_{N\times N}$, and N=|V| is the total number of nodes. In undirected and unweighted network, $a_{ij}^{t}=1$ when edge $e\left(i,j\right)\in E_{t}$ is connected, otherwise $a_{ij}^{t}=0$. When a set of network snapshots and their adjacency matrix sequences $\left\{A_{t-T},A_{t-T+1},\cdots ,A_{t}\right\}$ are given, the dynamic temporal link prediction method aims to study a function f(·) to predict the network adjacency matrix $A_{t+1}$ at the time *t*. It generally includes three steps:

1. Propose a new similarity index and its corresponding calculation function to calculate the similarity score of each snapshot;
2. Propose a new temporal evolution model and its corresponding learning function to predict the expected value in the future;
3. Compare and evaluate the expected prediction probability with the real topology.

(1) $$S_{t}=f_{t}\left(A_{t}\right)$$

(2) $$\overset{⌢}{A}=f_{2}\left(S_{t-T},S_{t-T+1},\cdots ,S_{t}\right)$$

(3) $$AUC=evaluate({\overset{⌢}{A}}_{t+1},A_{t+1})$$

**Figure 2:**
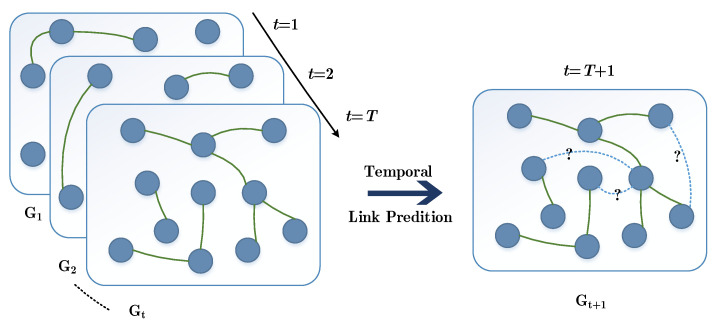
Network slicing instance deployment Diagram of temporal link prediction, which can predict the topology situation at T+1 from information of snapshot [1,⋯,T].

As shown in Figure 2, network *G* is the set of all nodes and edges of the dynamic network, $G_{t}=G\left(V_{t},E_{t}\right)$ represents the network snapshot at time *t*, *V* is the node set and *E* is the edge set at time *t*. Each node can contain multiple attribute information, and each edge can contain multiple weight information. Nodes and edges can dynamically increase and disappear in the temporal network. The problem of temporal link prediction is to predict the topology connection of the network at time *t* using the model trained by topology and attribute information of multiple snapshots before time *t*.

### 2.3. Metrics

Area under curve (AUC) [22] is a widely used metric for performance evaluation. The AUC measure gives values between 0 and 1, and values above 0.5 show that the proposed algorithm is better than the random prediction for binary classes. In terms of link prediction, AUC means that the probability of a randomly chosen actual edge score is higher than a randomly chosen nonexistent edge score. AUC score is calculated as given in Equation (4):

(4) $$AUC=\frac{n^{\prime }+0.5n^{\prime \prime }}{n}$$

where *n* is the times of independent comparisons, $n^{\prime }$ indicates how many times actual edge score is higher than nonexistent edge score, and $n^{\prime \prime }$ shows how many times scores of actual and nonexistent edges are equal.

## 3. TLP-CCC Algorithm

**Figure 3:**
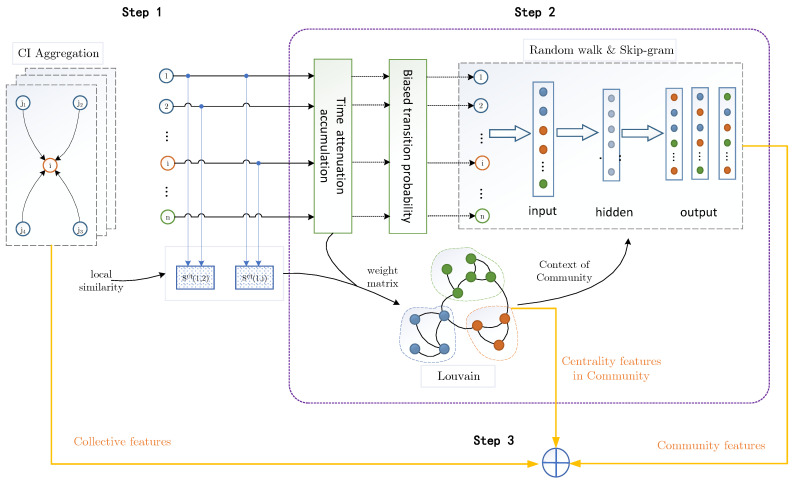
Diagram of the multi-features fusion and embedded representation for temporal link prediction. Step 1: Motivated by the concept of collective influence in percolation optimization theory, the collective influence is considered as the effective attribute of nodes, and construct the weight matrix of edges based on node attribute. Step 2: With CI-based weight matrix and weak link optimization, design the mechanism of node random walk within the community, then a novel strategy of next hop with priority to the existence of connected edges within the community, and the improved Skip-gram model is used to obtain the node representation vector. Step 3: Concatenate the collective influence, network centrality impact and the representation learning vectors of nodes. By using the joint new vectors to calculate the score matrix of the edges, and the temporal link prediction method TLP-CCC is proposed.

As shown in Figure 3, the proposed algorithm can be divided into three steps. Firstly, quantify node weight and edge weight by using collective influence. Secondly, after processes of time attenuation accumulation and biased transition probability, detect communities by Louvain algorithm according to the principle of modularity optimization, let the nodes perform supervised random walk in the context of the community, and the Skip-gram model is used to obtain the representation vector. Finally, the collective influence, degree centrality, closeness centrality, and betweenness centrality, are combined with the trained node representation vectors, and uses cosine similarity to calculate the similarity index $S^{CCC}$, and the temporal link prediction method TLP-CCC is based on $S^{CCC}$ index.

### 3.1. Similarity Index Based on Collective Influence

**Figure 4:**
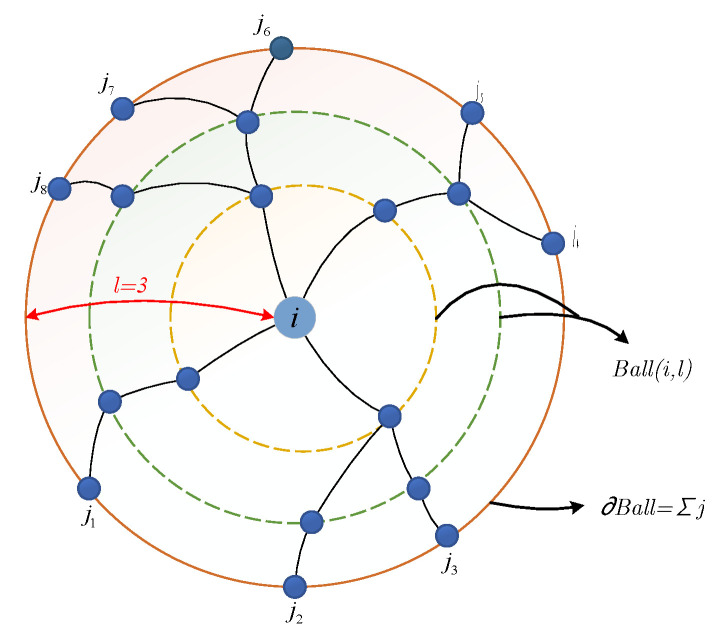
Diagram of the collective influence of node. The influence is determined by the joint importance of itself and nodes on the ball boundary.

The percolation optimal model is generally used to solve the critical problem of connectivity in complex networks. The optimization of the percolation model is to find the minimum node set that can destroy the maximum connected component of the network, or find a group of nodes that play an important role in the global connection of the network. To solve this problem, Morone et al. [23] proposed a Collective Influence algorithm to quantify the influence of nodes. The collective influence of a node is characterized by nodes on the spherical boundary and has nothing to do with other nodes on the inner path of the ball. The collective influence can more effectively quantify the topology information of nodes in the local range. As shown in Figure 4, the collective influence of nodes in the range of radius 3 can be characterized by boundary nodes $j_{1}$ to $j_{8}$ and node *i*. The collective influence of node *i* can be defined as:

The collective influence of node *i* can be defined [23] as:

(5) $$CI_{\ell }\left(i\right)=\left(k_{i}-1\right)\sum_{j\in \partial Ball(i,l)}\left(k_{j}-1\right)$$

where $k_{i}$ represents the degree of node *i*, *ℓ* is the radius of the ball, i.e., the path length from ball boundary node to center node, Ball(i,l) is the node set in the ball with node *i* as the center and *l* as the radius, ∂Ball=∑j represents the boundary of the ball. The CI algorithm can make more effective use of local topology information. In this paper, the radius *ℓ* is set to 3.

**Figure 5:**
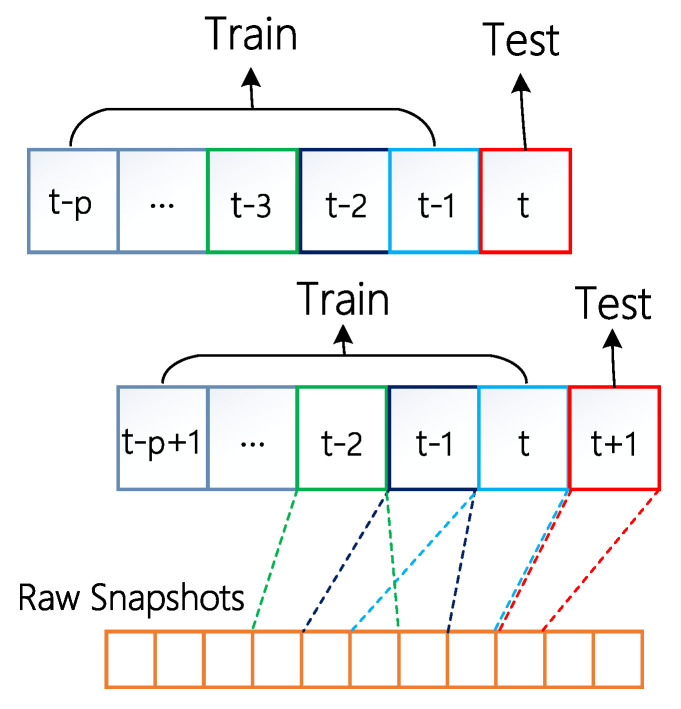
Schematic diagram of dynamic network data set division. First, construct every time unit from the sliding window. Second, split the data set into the training set and the testing set. Finally, move the train window and the test window step by step, to achieve the mean prediction evaluation.

The dynamic network can be divided into multiple snapshot sets according to a fixed time interval, the train set and the test set can be split as shown in Figure 5. When predicting the network topology at a certain time, *p* time sliding windows are required as the training set. In order to make full use of the time evolution information, the exponential function is used to fit the temporal evolution of the network to obtain the collective influence weight of each node in the training set.

(6) $$CI^{v}\left(i\right)=\sum_{t=T-p}^{T}\alpha^{t-T}\cdot CI_{\ell }\left(i,t\right)$$

where α represents the time attenuation parameter, and α>1. The greater the value, the smaller the impact of the snapshot relatively far from the prediction time, and $CI_{\ell }\left(i,t\right)$ represents the collective influence intensity of node *i* at time *t*.

After obtaining the collective influence of all nodes, the similarity index $S^{CI}$ based on the common neighbor index and the collective influence on node pair (i,j) is defined as Equation (7). Because CI is generally a value greater than 1, the numerator is the product of two nodes’ CI, which is used to quantitatively describe the influence of large nodes in social networks. The denominator is the sum of two CI, which indicates the average influence of the node on the surrounding edges. If there is only one edge between two nodes, the collective influence of the node pair is defined as 1.

(7) $$S^{CI}\left(i,j\right)=\left\{\begin{array}{cc} CN\left(i,j\right)\cdot \frac{CI^{v}\left(i\right)\times CI^{v}\left(j\right)}{CI^{v}\left(i\right)+CI^{v}\left(j\right)} & \mathrm{if}k_{i}>1\;\mathrm{and}\;k_{j}>1\\ 1 & \mathrm{if}k_{i}=1\;\mathrm{and}\;k_{j}=1 \end{array}\right.$$

### 3.2. Similarity Index Based on Subgraph Walk

The method in this section first divides subgraphs according to community classification, and then performs a random walk within the range of subgraphs.

Firstly, we construct weighted subgraphs using the edge collective influence obtained in the previous section. At the same time, considering that the weak correlation often affects the expression of the association degree between nodes, to filter particularly large and unreasonable communities when dividing large-scale networks, the minimum 5% value in the CI weight matrix with edges is replaced with 0, as shown in Equation (8).

(8) $$CI^{e}\left(i,j\right)=\left\{\begin{array}{cc} S^{CI}\left(i,j\right) & \mathrm{if}S^{CI}\left(i,j\right)>rank\left(5\%\right)\\ 0 & \mathrm{otherwise} \end{array}\right.$$

In order to ensure that the edge weight actually exists in the network, the CI weight matrix is correspondingly multiplied by the adjacency matrix to obtain the weight matrix of the collective influence of all edges. At this time, the adjacency matrix Adj is the cumulative weight matrix within the time step *p* of the training set.

(9) $$\begin{array}{c} M_{ij}=S^{CI}\left(i,j\right)\cdot Adj\left(i,j\right)\\ Adj\left(i,j\right)=\sum_{t}^{t+p}\alpha^{t-T}\cdot A_{t}\left(i,j\right) \end{array}$$

To divide the network into several subgraphs using the Louvain community detection algorithm [24], we input into the module the network topology information and edge weight matrix of the training set. Then, it calculates iteratively according to the mechanism of maximizing modularity. The calculation method of modularity is as follows:

(10) $$Q=\frac{1}{2m}\sum_{i,j;i\neq j}(M_{ij}-\frac{k_{i}k_{j}}{2m})$$

where $M_{ij}$ represents the actual weight of the edge from node *i* to *j*, $k_{i}=\sum_{j}M_{ij}$ represents the weight sum of all edges connected to node *i*, $m=\frac{1}{2}\sum_{i,j}M_{ij}$ represents the weight sum of all edges, $k_{j}/2m$ represents the probability of the connection between the node *j* and any node in the whole graph, $k_{i}k_{j}/2m$ represents the expected weight of the edges between node *i* and *j*, and the difference between $M_{ij}$ and $k_{i}k_{j}/2m$ represents the final gain.

The pseudo-code process of calculating the collective influence weight matrix and community division is shown in Algorithm 1.

Secondly, design a supervised random walk strategy, the context scope of node walking is limited to the bounds of the community subgraph. At the same time, the edge with a large weight is preferentially selected in the random walk according to the collective influence of all edges obtained from Equation (7) when selecting the next hop. The Equation (11) represents the probability of walking from node *i* to the neighbor node *j*. Different from Deepwalk and node2vec, next hop strategy gives priority to the connected edges. For the random walk path from node *i* to node *j*, it is preferred to walk to the neighbor node when the neighbor had a link with the node *j*, and other cases as same as node2vec method.

**Algorithm 1** 
$Create\;Community\;Graph\;with\;CI(g_{t},\tau ,\alpha ).$

**Input:** 
Network snapshot $g_{t}=\left\{G^{t-\tau +1},\ldots ,G^{t-1},G^{t}\}\right.$; Time step τ; Time attenuation parameter α;1:**for** j=1 to τ **do**2:    **for** $node_{i}$ in $G^{j}$ **do**3:        Establish the collective influence weight of all $node_{i}$ according to Equation (5)4:    **end for**5:    Accumulate the vertex weight according to Equation (6)6:
**end for**
7:Construct the weight matrix according to Equation (7)8:Remove the edge weight matrix of weak connection according to Equation (8)9:Construct cumulative weight matrix according to (9)10:Community subgraph Detect $G^{*}=Louvain\left(G,M\right)$**Output:** Community subgraph list $G^{*}$; Node Collective influence weight matrix $CI^{v}$; Edge Collective influence weight matrix $CI^{e}$;

(11) $$P\left(i|j\right)=\left\{\begin{array}{cc} \frac{CI(i,j)}{\sum_{z}CI\left(i,z\right)} & \mathrm{if}\;i,j\in C^{k},k=1,2,\cdots \\ 0 & \mathrm{else} \end{array}\right.$$

where *z* represents all adjacent nodes of node *i* in its community, $C^{k}$ represents the *k*-th community. Two nodes in the network belong to the same community, and the greater the degree of association between them, the greater the probability of biased random walk between them.

After obtaining the random walk sequence of each node, the node sequence can be input into the classical Skip-gram model to learn the representation vector of the node. Skip-gram is a natural language processing (NLP) model, which is used to maximize the co-occurrence probability between words in the window. It can predict the context node when the current node is known, that is, input the walking information of one node and output the representation vector of multiple nodes. In this section, the Skip-gram model is used to set the following objective functions combined with the divided subgraphs of each community to increase the co-occurrence probability:

(12) $$\varsigma =\max_{\theta }\sum_{i\in V}\log P(\Gamma \left(i\right)\left|i;\theta ,C\right.)$$

where *i*, Γ(i), *C*, θ represents the input node information, neighbor set of input node, community information, model parameters, respectively. θ is composed of two matrices *u* and *v*, and *u* is the context matrix and *v* is the node characteristic matrix. The optimal *u* and *v* are finally obtained through iterative training. $P(\Gamma \left(i\right)\left|i;\theta ,C\right.)$ can be also expressed as:

(13) $$P\left(\Gamma \left(i\right)\left|i;\theta ,C\right.\right)=\prod_{j\in \Gamma (i)}p(j\left|i;\theta \right.,C)$$

where $p(j\left|i;\theta \right.,C)$ represents the probability that neighbor node *j* exists in the walking sequence. Finally, the output result is optimized by softmax function, so that:

(14) $$p\left(j\left|i;\theta \right.,C\right)=\frac{e^{u_{j}}\dot{e^{v_{i}}}}{\sum_{z\in V}e^{u_{z}}\dot{e^{v_{i}}}}$$

where $u_{j}$ is the *j*-th row of *u* representing the context vector of neighbor node *j*, and $v_{i}$ is the *i*-th row of *v* to be regarded as the representation vector of seed node *i*. *z* represents other community nodes except the current context node. The conditional probability result of Equation (14) can be obtained by softmax on the inner product of the two vectors.

In order to improve the computational efficiency, the negative sampling method is used to optimize the model. The probability that a node is selected as a negative sample can be obtained from the degree distribution of the node, which can be expressed as follow according to Reference [25]:

(15) $$P\left(i\right)=\frac{f{\left(i\right)}^{\frac{3}{4}}}{\sum_{j=1}^{K}{\left(j\right)}^{\frac{3}{4}}}$$

where *K* is the number of negative samples, f(i) is the collective influence weight of node *i*. Therefore, based on the improved Skip-gram model and negative sampling method, the objective function of the algorithm can be updated as:

(16) $$O\left(X\right)=\log\delta \left(u_{j}\cdot v_{i}\right)-\sum_{k=1}^{neg}\delta (u_{k}\cdot v_{i})$$

where δ(x)=1/(1+exp(−x)) is the sigmoid function, $u_{j}$ represents the neighbor node vector information sampled in the community, $u_{k}$ represents the negative sample information in the community.

The more similar the two node vectors are, the greater the result of point multiplication as well as the probability value obtained after normalization. The normalized probability value of point multiplication can be used to represent the similarity degree of edges. The similarity index $S^{Com}$ based on community-biased embedding representation is defined as Equation (17), in which $X^{com\_emb}$ represents the embedded representation vector of all nodes after community division, biased walk, and Skip-gram learning.

(17) $$S^{Com}=\left\{\begin{array}{cc} X^{com\_emb}\cdot {\left(X^{com\_emb}\right)}^{T} & \mathrm{if}\;i\neq j\\ 0 & \mathrm{otherwise} \end{array}\right.$$

The pseudo-code of computing the embedded representation learning process based on the community-biased random walk is shown in Algorithm 2.

**Algorithm 2** 
$Sub\;Graph\;RW\;Skipgram(g_{t},\tau ,w,n,d,l).$

**Input:** 
Time step τ; Dimensions *d*; Walk length *l*; Num walks *n*; Window size *w*; Network snapshot $g_{t}=\left\{G^{t-\tau +1},\ldots ,G^{t-1},G^{t}\}\right.$;1:Initialize vector matrix X2:

$G^{*},CI^{v},CI^{e}=CreateCommunityGraphWithCI\left(g,T,\alpha \right)$

3:**for** com to $G^{*}$ **do**4:    **for** i=1 to *n* **do**5:        **for** $v_{i}\in com$ **do**6:           Calculate the probability transfer matrix **P** according to Equation (13)7:           $walks=BiasedRandomwalk(com,v_{i},d,l,P)$8:           X=Skipgram(X,w,walks)9:        **end for**10:    **end for**11:
**end for**
12: 13:Calculate $S^{Com}$ according to Equation (17)**Output:** Node Vector representation matrix $X\in R^{V\times d}$; Similarity index based on community biased embedding representation $S^{Com}$;


### 3.3. Similarity Index Based on Multi Feature Fusion

The node centrality index reflects the node importance in networks. Reference [26] makes effective use of the network structure information and proposes the similarity index fusing community and centrality index. Reference [27] proposes a subgraph similarity feature sequence integrating multiple local similarity indexes and weights. Inspired by these works, the node collective influence weight obtained by Equation (6) and the node subgraph centrality feature are fused with the $X^{ccc\_emb}$ representation vector in Equation (17) to obtain the new node representation vector, and then the similarity index $S^{CCC\_emb}$ of multi-feature vector fusion is calculated by cosine similarity.

The degree centrality feature is calculated only within the community to which the node belongs. As shown in Equation (18). α represents the exponential attenuation parameter. $k_{i}$ represents the degree of node *i*. *n* represents the number of all nodes in the network. *C* represents the community to which node *i* belongs.

(18) $$DC\left(i\right)=\sum_{t=T-p}^{T}\alpha^{t-T}\cdot \frac{k_{i}}{n-1}\;|\;k_{i}=\Gamma \left(i\right)\;\mathrm{and}\;i,\Gamma \left(i\right)\in C$$

The node betweenness centrality is calculated as Equation (19). α represents the exponential attenuation parameter. $\sigma_{st}$ represents the number of shortest paths of s→t. $\sigma_{st}\left(i\right)$ represents the number of shortest paths of s→t passing through node *i*, and *C* represents the community to which nodes s,i,j belong.

(19) $$BC\left(i\right)=\sum_{t=T-p}^{T}\alpha^{t-T}\cdot \sum_{s\neq t\neq i\in V}\frac{\sigma_{st}\left(i\right)}{\sigma_{st}}\;|\;s,i,t\in C$$

The node closeness is calculated as Equation (20). α represents the exponential attenuation parameter, *n* represents the number of all nodes in the network, d(i,j) is the average distance between node *i* and *j*, and *C* represents the community to which nodes i,j belong.

(20) $$CC\left(i\right)=\sum_{t=T-p}^{T}\alpha^{t-T}\cdot \frac{n-1}{\sum_{j-1}^{n-1}d(i,j)}\;|\;i,j\in C$$

Afterward, the new node representation vector obtained by multi feature fusion is shown in Equation (21), and the features are directly connected with each other to form the new vector.

(21) $$X^{ccc\_emb}=\left[DC:BC:CC:CI,X^{com\_emb}\right]$$

Cosine similarity measures the similarity of two vectors by calculating the cosine value of the angle between two vectors. For *d*-dimensional vectors A and B:

(22) $$\begin{array}{c} S^{CCC}=\mathrm{cosine}(X^{ccc\_emb},X^{ccc\_emb})\\ \mathrm{cosine}(A,B)=\frac{\sum_{1}^{n}\left(A_{i}\times B_{i}\right)}{\sqrt{\sum_{1}^{n}A_{i}^{2}}\times \sqrt{\sum_{1}^{n}B_{i}^{2}}} \end{array}$$

The range of cosine similarity is between [−1,1]. The larger the value which represents the high similarity, the smaller the angle between the two vectors. The smaller the value which represents the low similarity, the greater the angle between the two vectors.

The pseudo-code of the temporal link prediction method based on multi-feature fusion embedded representation is shown in Algorithm 3.

In Algorithm 3, the model estimation in step 5 requires time depending on the Louvain method, which is a fast algorithm, and most of the computational time is exploited by step 6, which is determined by the efficiency of step 7 and step 8 of Algorithm 2. For the random walk, the time complexity is O(ln|V|+2|E|), where *n* is walk num, *l* is walk length. For the skip-gram, the time complexity is O(|V|·|V|), and it can be optimized to O(2dw|V|), where *d* is dimensions and *w* is the window size. Therefore, the time complexity of the proposed method is O((ln|V|+2|E|)·(dw|V|)), that is $O(lndw|V{|}^{2})$ finally.

**Algorithm 3** Multifeaturepredict(G,size).
**Input:** 
Network snapshot $G=\left\{G^{1},G^{2},...,G^{t-1},G^{t}\}\right.$; Train window size size; Window size *w*; Num walks *n*; Dimensions *d*; Walk length *l*;1:Initialize score matrix list Score_auc2:Initialize vector matrix X_CCC3:**for** τ in [size,G.len−1] **do**4:    Construct the train snapshots list $g_{t}=G\left(\tau \right)$5:    $G^{*},CI^{v},CI^{e}=CreateCommunityGraphWithCI\left(g_{t},\tau ,\alpha \right)$6:    $X,...=SubGraphRWSkipgram(G^{*},w,n,d,l)$7:    Calculate DC,BC,CC according to Equation (18) ∼ Equation (20)8:    $X\_CCC=Concat(DC,BC,CC,CI^{v},X)$ according to Equation (21)9:    Calculate $S^{CCC}$ according to Equation (22)10:    $auc=evaluate(G^{\tau +1},S^{CCC})$11:    Score_auc.append(auc)12:
**end for**
13:

mean_auc=mean(Score_auc)

**Output:** Average AUC result mean_auc;


## 4. Experiment

### 4.1. Datasets

In this paper, nine communication network data sets are used to evaluate the performance of the algorithm. Email [28] is generated from the email data of a large European research institution. Enron [29] is composed of email data sent between Enron employees. Facebook [30] contains the exchange records of Facebook users leaving messages on another user’s wall. DNC [31] is an email exchange network collected by the Democratic National Committee in the event of email leakage. Man [31] is the mail record of employees in a manufacturing factory. UCI [32] is an online social network composed of text messages transmitted between students at the University of California, Irvine. LEM [33] is an interactive network collected by the Kansas event data system based on folders containing WEIS coded events, covering events from April 1979 to June 2004. BIT [32] is a record of reputation scores among members in the Bitcoin OTC trading platform. The SXA2Q [34] is a record of interactions on the stack exchange website Ask Ubuntu. Basic characteristic parameters of data sets are shown in Table 1.

### 4.2. Baselines

The algorithms compared in this paper include methods based on moving average, topology trend change, and node importance indices. The training and testing set sizes of baseline methods are the same as the proposed method. Details are as follows:

- Moving Average [35]: this kind of method makes quantitative analysis by using the similarity mean of relevant snapshots within the moving range, as shown in Formula (23), which is recorded as Average. If the moving range is only one window before prediction, the model can be used as the nearest time algorithm, as shown in Equation (24), which is recorded as Last. If the whole moments of the moving range are regarded as a weighted static window, the model can be used as the nearest neighbor algorithm of the iteration cycle, as shown in Equation (25), which is recorded as Reduce.
  (23) $${\overset{⌢}{\;S}}^{\mathrm{Average}}(T)=\frac{1}{m}\sum_{t=T-m-1}^{T-1}f_{2}\left(A(t)\right)$$
  (24) $${\overset{⌢}{\;S}}^{\mathrm{Last}}(T)=f_{2}\left(A(T-1)\right)$$
  (25) $${\overset{⌢}{\;S}}^{\mathrm{Reduce}}(T)=f2(\sum_{t=T-p+1}^{T-1}A(t))$$
- Node2vec [36]: this method uses the idea of word embedding, inputs the network topology and outputs the representation vector of each node. Equation (26) is the objective function, f(u) is the node mapping function, $N_{s}\left(u\right)$ is the characteristic node set of node *u* sampled by sampling strategy *S*. In this paper, we set the walking parameters p=1 and q=2, and give priority to the breadth walking strategy.
  (26) $$\max\sum_{u\in V}\left[-\log Z_{u}+\sum_{n_{i}\in N_{s}\left(u\right)}f(n_{i})\cdot f\left(u\right)\right]$$
- LINE [37]: this method uses the first-order information directly connected between nodes and the second-order information of common neighbors to jointly design the objective function, such as Equation (27), $O_{1}$ and $O_{2}$ are the first-order and second-order objective function, respectively, $w_{ij}$ is the edge weight. This paper uses the second-order similarity method for calculation.
  (27) $$\begin{array}{c} O_{1}=-\sum_{(i,j)\in E}w_{ij}\log p_{1}\left(v_{i},v_{j}\right)\\ O_{2}=-\sum_{(i,j)\in E}w_{ij}\log p_{2}\left(v_{j}|v_{i}\right) \end{array}$$
- DySAT [38]: this method combines the structural self-attention layer characteristics and temporal self-attention layer characteristics of the network. The multi-head attention mechanism is adopted to capture the evolution characteristics between network snapshots. The obtained graph embedded representation vectors are used to realize link prediction. Equation (28) is the loss function of the prediction model.
  (28) $$L=\sum_{t=1}^{T}\sum_{v\in V}\left(\sum_{u\in N_{walk}^{t}\left(v\right)}-\log(\sigma \left(<e_{u}^{t},e_{v}^{t}>\right)-w_{n}\cdot \sum_{u^{\prime }\in P_{n}^{t}\left(v\right)}\log(1-\sigma \left(<e_{u}^{t},e_{v}^{t}>\right)\right))$$
- TSAM [39]: This method uses graph attention network to capture network motif features, and gated recurrent units (GRU) are utilized to learn temporal variations in the snapshot sequence. Both node-level self-attention and time-level self-attention mechanisms are adopted in the model to accelerate the learning process and improve the prediction performance. Equation (29) is the loss function of the prediction model.
  (29) $$L_{t}\left(\theta ;A_{t-T}^{t},A_{t+1}\right)={∥\left(S_{t+1}-A_{t+1}\right)⊙B∥}_{F}^{2}+\frac{\lambda }{2}{∥\theta ∥}_{2}^{2}$$
- EvolveGCN [40]: this method uses gated recurrent unit (GRU) or long short-term memory network (LSTM) to adjust the parameters of graph convolutional network (GCN) at each time step to capture the dynamic characteristics of graph sequence and then realize the prediction function. This paper uses LSTM to dynamically adjust the network parameters.
  (30) $$\begin{array}{c} H_{t}^{\left(l+1\right)}=GCONV\left(A_{t},H_{t}^{\left(l\right)},W_{t}^{\left(l\right)}\right)\\ W_{t}^{\left(l\right)}=GRU/LSTM\left(W_{t-1}^{\left(l\right)}\right) \end{array}$$

### 4.3. Results

#### 4.3.1. Comparison of Link Prediction Accuracy under AUC Standard

In Experiment 1, set the sphere radius of collective influence ℓ=3, that is, the points on the boundary of the sphere with radius 3 are considered for calculation. Set the training parameters sliding window lag=1, time window T=7, that is, using the network data of 7 time steps before the prediction time for training. Set the temporal attenuation coefficient of collective influence α=0.9, and set the random walk parameters, including the dimensions = 128, the walk-length = 80, the num-walks = 10, the window-size = 10, and concatenate the node collective influence, the three centrality influence, and the walking embedded vector to predict by using the similarity score of Equation (24), which is recorded as TLP-CCC. The AUC performance comparison of the experimental results is shown in Table 2, TLP-CCC method achieves the best prediction accuracy in all data sets.

Firstly, compared to the proposed method with the three CN-based moving average temporal methods, namely Last, Average, and Reduce, it can be seen that the prediction performance of Last is the worst, Average is better than Last and worse than Reduce, which indicates that more topology information can improve the prediction accuracy. These three methods perform well in MAN and LEM datasets, but are a little worse than TLP-CCC. For the other seven datasets, the mean performance of TLP-CCC has been greatly improved, which is about 39% higher than last, 23% higher than Average and 17% higher than Reduce.

Then, we compared the network representation learning methods of node2vec and LINE. These two methods are not end-to-end learning, and the learning process of the node vector is closely related to the random walk path. Useful information in local topology cannot be accurately captured in many cases, and the prediction accuracy will fluctuate by about 1%. TLP-CCC performs a little better in Enron and Facebook, about 2% higher than node2vec and 3% than LINE. For the other seven datasets, the AUC value of TLP-CCC is increased by 11∼49%.

Moreover, compared with the DySAT, TSAM, and EvolveGCN methods based on graph neural network. The results show that the graph neural network methods use richer data dimensions for training and establishes more hidden layer networks to represent the topology, but the improvement in the actual prediction is limited. The accuracy of TLP-CCC has slightly improved in MAN dataset and 7∼26% higher in the other eight datasets.

In a word, methods based on static indices moving average perform better in small scale network, while methods based on graph neural network are relatively more adaptable for large scale complex network applications, and TLP-CCC is outperforming better in nine datasets.

#### 4.3.2. Sensitivity Test of Ball Radius Parameters

**Figure 6:**
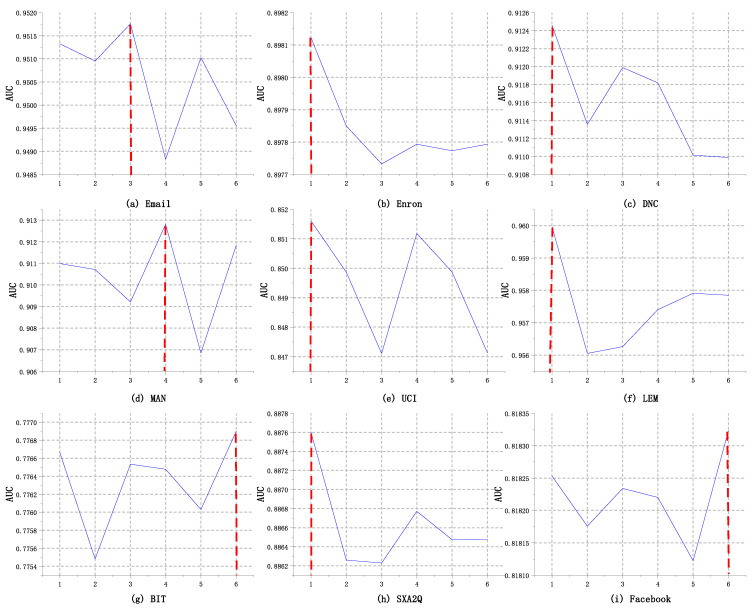
Comparison results of different collective influence radius. (**a**) The optimal radius of the Email data set is 3. (**b**) the optimal radius of the Enron data set is 1. (**c**) the optimal radius of the DNC data set is 1. (**d**) the optimal radius of the MAN data set is 4. (**e**) the optimal radius of the UCI data set is 1. (**f**) the optimal radius of the LEM data set is 1. (**g**) the optimal radius of the BIT data set is 6. (**h**) the optimal radius of the SXA2Q data set is 1. (**i**) the optimal radius of the Facebook data set is 6.

The reference [23] recommends setting a three-layer radius in the experiment of connectivity optimization, we set the layer number of the collective influence method from 1 to 6, and observe the influence of the ball radius for the TLP-CCC AUC value. As shown in Figure 6, in the five datasets of Enron, DNC, UCI, LEM, and SXA2Q, the AUC value curve has the largest value when the radius is 1. With the increase of radius, the AUC results show a small fluctuation trend. In BIT and Facebook, it can get the best AUC value when the radius is 6, and in Email and MAN, the best AUC value when the radius is 3 or 4. These results show that using the topology information of the nearest neighbor is the best for the temporal prediction of the structure.

To sum up, the layer number of the spherical radius has a little impact on TLP-CCC, and the fluctuation range of accuracy is about 1%. In general, when the radius of collective influence is large, the node-local topology information has certain limitations. The improvement effect of using only the information of collective influence on link prediction is limited. According to the experimental results, it is recommended to set the ball radius parameter to 1.

#### 4.3.3. Sensitivity Test of Random Walk Parameter

**Figure 7:**
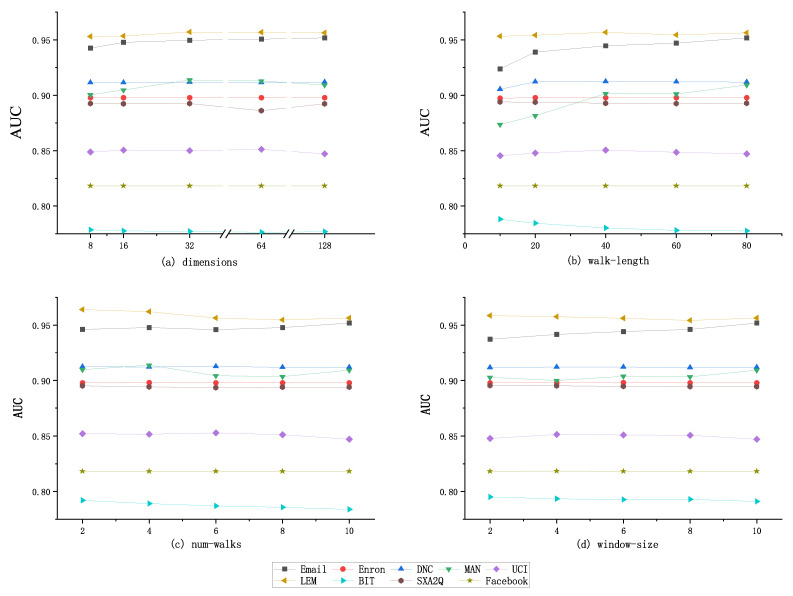
Prediction results of different walking parameters. (**a**) Different dimensions for nine data sets. (**b**) Different walk-length for nine data sets. (**c**) Different num-walks for nine data sets. (**d**) Different window-size for nine data sets.

The sensitivity of Skip-gram walk parameters is tested by observing the change of AUC score when changing the size of vector dimension, random walk step, node walk times, and walk window size. Figure 7 shows the score of link prediction using node representation vector when different settings of random walk parameters of TLP-CCC. Figure 7a shows the results of vector dimension parameters. With the increase of dimension parameters, the AUC score fluctuates in a small range, and the optimal value is obtained when the dimension is 32, 64, or 128, respectively. However, the change of dimension parameters has little impact on the AUC score, and the fluctuation range is about 1%. Figure 7b shows the results of the walking step parameter. With the increase of the parameter, the AUC score also gradually increases in a small range. We can see that it has a great impact on the Email and MAN datasets, which increases by about 3% when the step is 80 compared with 20, and the change of the other 4 datasets does not exceed 1%. Figure 7c,d show the test results of the changes of the walk number parameter and the walk window. With the increase of the parameter, the AUC score changes slightly. Considering the computational complexity and the experimental average effect, it is recommended to set the vector dimension greater than 64, the walk step between 40 and 80, the walk times to be 5 and the walk window size to be 10.

#### 4.3.4. Sensitivity Test of Training Window Size

**Figure 8:**
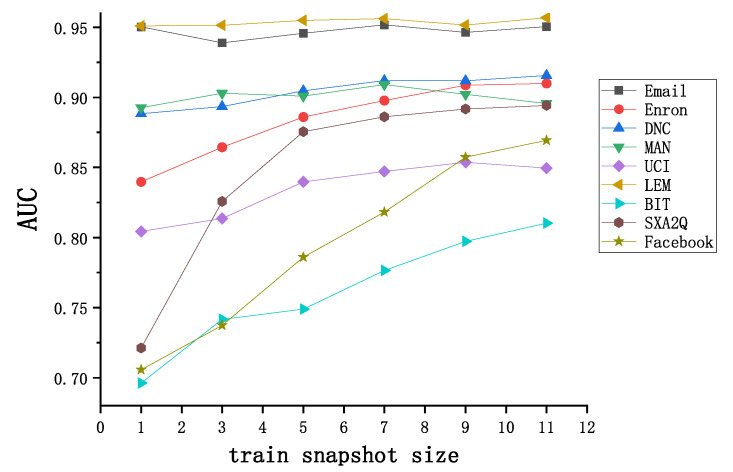
AUC performance results of different train window size.

For different data sets, with the training snapshot window size set to 1, 3, 5, 7, 9, and 11, respectively, the experimental results are shown as Figure 8. When the train window size becomes larger, TLP-CCC changes linearly in most datasets except Email, MAN, and UCI, and the optimal value is obtained when the window size is 7 or 9 in the other 3 datasets, which indicates that the topology information of the snapshot will have a certain negative impact on the link prediction result when the initial time is far from the prediction time. Generally speaking, the larger the training window, the better the prediction accuracy in most cases, but there are also fluctuations with the change of the window. Therefore, it is necessary to select the appropriate window size for different real networks.

## 5. Conclusions

Combining with sociological theory, this paper proposes three novel similarity indices, including $S^{CI}$ based on collective influence, $S^{Com}$ based on community biased walk, and $S^{CCC}$ based on multi-feature fusion. The novel method TLP-CCC uses collective influence, degree centrality, betweenness centrality, closeness, and representation learning within the community, which can make more effective use of network subgraph structure information and dynamic network evolution information. By comparing several temporal link prediction methods including moving average, network representation learning, and graph neural network, the experimental results show that our proposed method achieved better prediction accuracy and robustness in nine datasets. This paper does not analyze the impact of the interaction between different subgraphs on the prediction results. The follow-up research problem in the future is how to characterize the different roles of nodes in subgraphs and the influence between subgraphs.

## Figures and Tables

**Table 1:** Basic characteristic parameters of dataset.

Dataset	Email	Enron	Facebook	DNC	MAN	UCI	LEM	BIT	SXA2Q
Node number	1005	87,273	60,290	2029	167	1899	485	5881	137,517
Edge number	332,334	1,048,576	838,090	39,264	82,927	59,835	196,364	35,592	280,102
Start date	October 2003	1 January 2001	November 2006	23 April 2016	January 2010	15 April 2004	April 1979	9 November 2010	29 September 2009
End date	May 2005	31 March 2002	January 2009	25 May 2016	October 2010	26 October 2004	June 2004	25 January 2016	6 March 2016
Total duration	526 days	454 days	103 weeks	33 days	268 days	195 days	303 months	1904 days	2351 days
Temporal period	week	week	2 weeks	day	week	week	half a year	month	month
Snapshot number	76	66	52	33	39	28	51	63	79

**Table 2:** Comparison results of prediction accuracy AUC. The proposed method achieves the best prediction accuracy in nine data sets.

Dataset	Email	Enron	Facebook	DNC	MAN	UCI	LEM	BIT	SXA2Q
Last	0.7535	0.6056	0.5158	0.7011	0.8415	0.5152	0.9021	0.5376	0.5340
Average	0.8793	0.7282	0.5350	0.8156	0.8964	0.5724	0.9520	0.5802	0.6604
Reduce	0.8954	0.7732	0.5522	0.8252	0.9025	0.6157	0.9530	0.6329	0.7593
node2vec	0.8384	0.8877	0.8049	0.8420	0.6028	0.5227	0.7444	0.7019	0.7981
LINE	0.8253	0.8784	0.7576	0.8624	0.6068	0.6507	0.7713	0.6266	0.7621
DySAT	0.8603	0.7885	0.7515	0.8376	0.9020	0.7187	0.8352	0.7173	0.8256
TSAM	0.8501	0.7945	0.7702	0.8462	0.8975	0.7230	0.8373	0.6534	0.7266
EvolveGCN	0.7503	0.6347	0.6752	0.8789	0.8016	0.6810	0.8969	0.7565	0.8098
TLP-CCC	**0.9518**	**0.9077**	**0.8182**	**0.9120**	**0.9092**	**0.8471**	**0.9563**	**0.7765**	**0.8865**

## Data Availability

Data, models, or code that support the findings of this study are available from the authors upon reasonable request.
